# A New Subspecies Identification and Population Study of the Asian Small-Clawed Otter (*Aonyx cinereus*) in Malay Peninsula and Southern Thailand Based on Fecal DNA Method

**DOI:** 10.1155/2014/457350

**Published:** 2014-03-13

**Authors:** M. K. A. Rosli, S. M. F. Syed-Shabthar, P. Abdul-Patah, Z. Abdul-Samad, S. N. Abdul, M. N. Burhanuddin, N. A. Zulkifli, M. N. Shukor, K. Budsabong, S. Changtragoon, T. Sekiguchi, H. Sasaki, B. M. Md-Zain

**Affiliations:** ^1^School of Environmental and Natural Resource Sciences, Faculty of Science and Technology, Universiti Kebangsaan Malaysia, 43600 Bangi, Selangor, Malaysia; ^2^Department of Wildlife and National Parks, Km 10, Jalan Cheras, 50664 Kuala Lumpur, Malaysia; ^3^Department of National Park, Wildlife and Plant Conservation Office, Ladyao, Chatuchak, Bangkok 10900, Thailand; ^4^Department of Molecular Biology, Graduate School of Medical Science, Kyushu University, 3-1-1 Maidashi, Higashi-ku, Fukuoka 812-8582, Japan; ^5^Chikushi Jogakuen University Junior College, 2-12-1 Ishizaka, Dazaifu 818-0192, Japan

## Abstract

Three species of otter can be found throughout Malay Peninsula: *Aonyx cinereus*, *Lutra sumatrana*, and *Lutrogale perspicillata*. In this study, we focused on the *A. cinereus* population that ranges from the southern and *the* east coast to the northern regions of Malay Peninsula up to southern Thailand to review the relationships between the populations based on the mitochondrial D-loop region. Forty-eight samples from six populations were recognized as Johor, Perak, Terengganu, Kelantan, Ranong, and Thale Noi. Among the 48 samples, 33 were identified as *A. cinereus*, seven as *L. sumatrana*, and eight as *L. perspicillata*. Phylogenetically, two subclades formed for *A. cinereus*. The first subclade grouped all Malay Peninsula samples except for samples from Kelantan, and the second subclade grouped Kelantan samples with Thai sample. Genetic distance analysis supported the close relationships between Thai and Kelantan samples compared to the samples from Terengganu and the other Malaysian states. A minimum-spanning network showed that Kelantan and Thailand formed a haplogroup distinct from the other populations. Our results show that Thai subspecies *A. cinereus* may have migrated to Kelantan from Thai mainland. We also suggest the classification of a new subspecies from Malay Peninsula, the small-clawed otter named *A. cinereus kecilensis*.

## 1. Introduction

Several methods for identifying species rely on DNA sequence analysis. Among them are RFLPs, AFLPs, RAPD, polymerase chain reaction, microarray, and DNA sequencing [1–4]. DNA-based methods are often used due to the reliability of DNA sequences, with the general assumption that individuals from the same species carry specific DNA (or protein) sequences that differ from those found in individuals from other species [5]. DNA is a strong tool in forensic analysis because DNA is extremely stable and lives long as a biological molecule that can be recovered from several types of forensic evidence such as bloodstains, feces, saliva, urine, and commercial products [6, 7].

DNA extraction from feces is among the most complicated and high-risk processes of noninvasive samples. After DNA has been extracted, problems are often encountered in terms of relatively low DNA yields and/or recovering DNA free of inhibitory substances [8]. These issues make fecal sampling less popular among geneticists. However, fecal samples are important in studies that require DNA sources from risky or highly endangered subjects before samples are collected in the wild [4, 9]. With several nonconventional DNA extraction kits and enhanced PCR chemicals available today, DNA analysis of fecal sources is no longer impossible [10]. In this study, we focused on extracting DNA from fecal samples that are believed to be otter feces, collected from several regions, to identify species.

Otters belong to the mammalian order under the family Mustelidae and subfamily Lutrinae [11]. Thirteen otter species are found in the world, comprising seven distinct genera made up of three major clades based on nuclear and mitochondrial markers [12, 13]. The three clades are the sea and river otters of Eurasia and Africa (*Aonyx*,* Enhydra*,* Hydrictis*,* Lutra*, and* Lutrogale*), the marine and river otters from North, Central, and South America (*Lontra*), and a basal lineage of* Pteronura brasiliensis* [12, 14, 15].

In Malaysia, three species of Eurasian otter can be found throughout Malay Peninsula:* Aonyx cinereus*,* Lutra sumatrana*, and* Lutrogale perspicillata* [11, 16, 17]. The* L. perspicillata* and* A. cinereus* species are common in Malay Peninsula, where they are widely distributed, while* L. sumatrana* is rare but apparently still found in the eastern Malay Peninsula [18]. Based on previous systematic studies of morphological data,* Lutra* and* Lutrogale* are often grouped together in Lutrini clade, distinct from* Amblonyx*, which is grouped with* Aonyx* in Aonychini clade [19–21]. However, molecular data has indicated that* L. perspicillata *is a sister clade to* A. cinereus* in a higher clade distinct from* L. sumatrana*, a species previously closely related to* L. perspicillata* morphologically [14].

Among the three species,* A. cinereus* is the smallest, with an estimated average body size of less than 3.5 kg and measuring about 652–939 mm [11, 22, 23]. Morphologically,* A. cinereus* is characterized by a small head, a neck larger than the head, short legs, and a flat tail [21]. Their paws are very dexterous. The body color is light brown with greyish white color on the lips, chin, and sides of the neck [22]. Body size and smaller claws distinguish the* A. cinereus* from other otter species [22, 24].

Otters are adapted for a semiaquatic life and are bioindicators of wetland ecosystems [25]. A recent study showed that* A. cinereus* is considered “vulnerable” according to the IUCN Red List Criteria due to declining populations resulting from habitat loss and land exploitation [11].* A. cinereus* has also been recognized as being “under local scrutiny” by the IUCN Otter Specialist Group for the Conservation of Nature [25]. Several studies reported the extent of the habitat losses occurring in South and Southeast Asia, and increased poaching has led to population reduction in many parts of the species' range [11, 26, 27]. In the wild,* A. cinereus* is probably the species most threatened by habitat destruction and pollution of the environment, especially due to chemical organochlorine [28]. These species are also killed for their skin, fur, and organs, which are believed to have medicinal value in Asia [28]. To date, studies investigating the population of* A. cinereus* are lacking, and nothing has been done specifically regarding the population reduction of this species.

Globally,* A. cinereus *is widely distributed from India throughout South Asia across Bangladesh, Myanmar, Thailand, Indonesia, and Malaysia and from Southeast Asia to the Philippines, Taiwan, and eastern and southern China in the north [11]. However, this small species is possibly extinct in Hong Kong and Singapore [28, 29].* A. cinereus* inhabits coastal habitats and inland rivers, swamps, mangroves, and paddy fields up to 2000 m above sea level [30, 31]. This species often inhabits areas close to human activity [31].* A. cinereus* coexists with* L. sumatrana* and* L. perspicillata* in many locations, including several rivers in Thailand and Malaysia [32]. Although all three species feed on the same prey, most* A. cinereus* members are crab eaters, while most of the other species feed on fish [33]. Coexistence is controlled by the selection of different habitats and food. In Southeast Asia,* A. cinereus* is abundant in irrigation canals and rice fields where* L. sumatrana* is not present [16]. On Malay Peninsula,* A. cinereus* is limited to small rivers and irrigation canals [34].

Geographic range and population studies are important to obtain information for requirements concerning ecology and conservation efforts to preserve species threatened with extinction [35]. In this phylogeography study, we focused on the* A. cinereus* population ranging from the southern and eastern coast to northern regions of Malay Peninsula up to southern Thailand to review the knowledge about the relationship between populations of this species using genetic methods. Species identified from fecal samples help us determine the species in sampling locations and the coexistence of multiple species. Our region of interest was the mitochondrial DNA, D-loop control region. The mitochondrial genome has been extensively used to amplify many genes of interest for phylogenetic studies [36–39]. Sequence divergence accumulates more rapidly in mitochondrial DNA (mtDNA) than in nuclear DNA due to the faster mutation rate and lack of repair system in mtDNA, which means that it often contains high levels of information variation [40]. The D-loop is a highly variable noncoding control region that has the highest polymorphism rate among mitochondrial genes and is widely used in genetic population studies [9, 41–43].

## 2. Materials and Methods

### 2.1. Sampling Locations, Samples, and DNA Isolation

**Figure 1:**
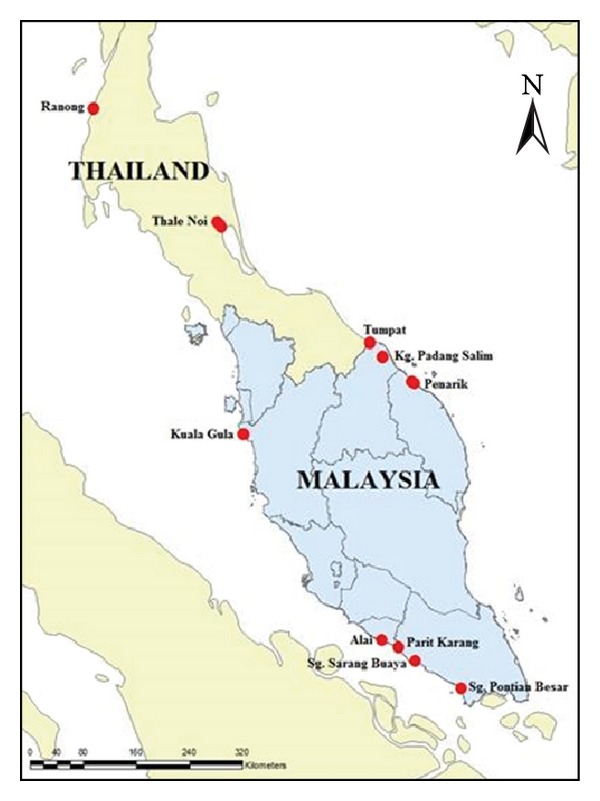
Sampling location of otter samples used in this study.

Genetic sampling involved several locations in Malay Peninsula including the northern region (Perak: Kuala Gula), the southern region (Johor: Sungai Pontian Besar, Parit Karang, and Sarang Buaya), and the eastern coastal region (Kelantan: Padang Salim, Tumpat; Terengganu: Penarik) of Malaysia and the southern region of Thailand (Ranong and Thale Noi) (Table 1). In this preliminary study, sampling locations were selected based on several reports of the existence of otters either by local people or parties, mainly at industrial fish ponds, lakes, and riversides within the same period of time. Forty-eight samples from six populations were recognized as Johor, Perak, Terengganu, Kelantan, Ranong, and Thale Noi in this study. Only the identified* A. cinereus* species samples are listed in Table 1.  Figure 1 summarizes the sampling location of this study. All samples were obtained with the help of various parties such as the Department of Wildlife and National Parks (Peninsular Malaysia), zoos in Malaysia, the Museum of Zoology (Universiti Kebangsaan Malaysia, UKM), Phnom Tamao Zoological Park and Wildlife Rescue Center, and Wakayama Adventure World. Fecal samples were used in this study. All samples were preserved in a TRIS, EDTA, ethanol, and/or SDS solution to prevent damage. The samples were kept in a refrigerator at –20°C or –80°C. Total genomic DNA was extracted by using the standard extraction kit and the protocol provided by the Qiagen DNeasy Stool Kit [44].

### 2.2. DNA Amplification

Polymerase chain reaction (PCR) was performed using a 25 *μ*L reaction mixture containing 1 *μ*L of genomic DNA, 2.5 *μ*L PCR Buffer 10X, 1 *μ*L 50 mM MgCl_2,_ 0.5 *μ*L 10 mM dNTP mix, 1.5 *μ*L each of 10 pmol/*μ*L primer, and 4 units of* Taq* DNA Polymerase in PTC-100 Thermal Cycler (MJ Research Inc.). The partial D-loop fragment of approximately 450 base pairs was amplified using forward primer TANA-B (5′-CGA AGC TTG ATA TGA AAA ACC ATC GTT G-3′) and reverse primer TANA-A (5′-GGA ATT CAT CTC TCC CGG TTT ACA AGA C-3′) [45]. PCR conditions were as follows: 4 min denaturation at 94°C, followed by 30 cycles of 30 sec at 94°C, 30 sec at 56°C, 1 min at 72°C, and a final 7 min extension at 72°C, before cooling to 4°C for 10 min. DNA from PCR products was purified using the Vivantis G-F1 PCR Clean-up Kit and was sent directly to the sequencing service company, First Base Sdn. Bhd., to be sequenced.

### 2.3. Species Identification and Phylogenetic Analysis

Sequencing results were exported as FASTA sequence files. Sequences from GenBank were obtained as positive controls for each species before the species was identified. The D-loop sequences of the studied samples were aligned using the ClustalW multiple alignment algorithm of BioEdit, together with control sequences from GenBank and an outgroup sequence of the palm civet. Identification at the species level was detected by informative polymorphic sites assigned to each species. All sequences were analyzed using PAUP 4.0b10 and MrBayes 3.1 for phylogeny reconstruction. Two methods of analysis in PAUP included the following: (1) neighbor-joining (NJ) with Kimura 2 Parameter [46], which takes into account the unequal rates of evolution of transition and transversion but assumes an equal distribution of nucleotide composition and (2) Maximum Parsimony (MP) with stepwise addition (1000 replicates) in heuristic search [47] and 50% majority rule consensus. In Maximum Parsimony (MP), gaps are treated as missing data, transitions and transversions are weighted equally, and heuristic search is performed with the TBR branch-swapping algorithm. All trees were subjected to bootstrap analysis with 1000 replicates to get bootstrap value support.

### 2.4. Genetic Structure of* A. cinereus*

Measures of population genetic parameters such as genetic diversity, nucleotide diversity, and nucleotide divergence among populations after nucleotide diversity was accounted for within populations (*D*_*a*_) were estimated from the mtDNA dataset using DNASP 4.0 [48]. The demographic history was examined with Tajima's test of neutrality, *D* test [49], and Fu's statistics test [50], to test for deviation in sequence variation from evolutionary neutrality. The tests compare the number of singleton mutations to the total number (*D*) and the average number of nucleotide differences between pairs of sequences (*F*), both under a neutral model [51].

Population bottlenecks and expansions, selective sweeps on the mtDNA, and mutational rate heterogeneity may all result in a Poisson distribution of substitution differences between pairs of haplotypes [52]. Therefore, mismatch distribution analysis was performed using Arlequin version 3.0 with 1000 permutations [53] and site-frequency spectra [54] as implemented in DNASP 4.0.

The population genetic structure was analyzed for samples with five or more individuals, with an analogue of Fst, Ost, as implemented in an analysis of molecular variance AMOVA [55] in Arlequin 3.0. The statistical significance was tested using 1000 permutations. The parsimony criterion was used to reconstruct the haplotype relationships of* A. cinereus*, assuming that differences at any given site between two randomly drawn haplotypes were unlikely to have arisen from more than 1 mutational step [56]. A minimum-spanning network was generated using Network 4.5.0.2 [57] to illustrate this relationship.

## 3. Results

**Figure 2:**
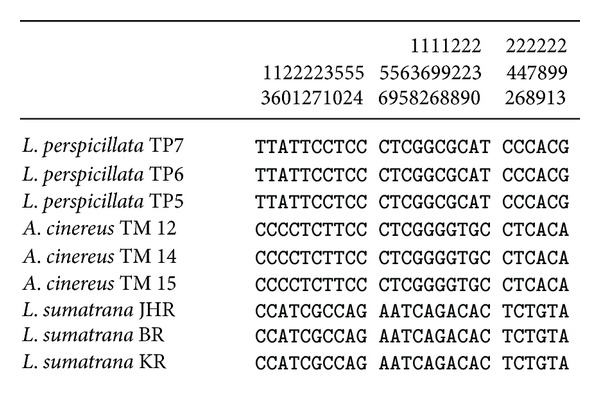
Polymorphic sites for* L. perspicillata*,* A. cinereus*, and* L. sumatrana* used to identify the species.

DNA from all 48 fecal samples was successfully extracted and sequenced. The partial D-loop sequences (398 bp) were converted into the FASTA format and were aligned with our control sequences of the three species* L. perspicillata*,* A. cinereus*, and* L. sumatrana* to identify species based on site polymorphisms. Based on the primers used in this study, several specific polymorphism sites were detected for each species including deletions and an insertion.  Figure 2 shows the informative polymorphic sites for all three species used for species identification in this study. Thirty informative sites were observed from the 398 bp sequence data set of the three species. Deletions and an insertion occurred specifically in the* L. sumatrana* species: deletions at site numbers 46–50 (134–136 at the full length sequence sites) and an insertion at site number 134 (201 at the full length sequence site). Among the 48 samples, 33 were identified as* A. cinereus*, seven as* L. sumatrana*, and eight as* L. perspicillata*. They are listed in Table 1.

In the sequence analysis of the 33* A. cinereus* samples, out of 405 bp partial sequences, 371 characters are conserved sites, and 34 characters are variable sites. Among the variable characters, 19 are singletons, and 15 characters are parsimony informative. The analysis showed that 8.4% of the total lengths are variable sites and about 3.7% are parsimony informative sites. The nucleotide compositions for the entire 405 bp sequences are as follows: *T* = 29.5%, *C* = 30.6%, *A* = 24%, and *G* = 15.9%.

Among the 33 individuals sequenced, 7 haplotypes were identified. Johor had two haplotypes (Hap 7 and Hap 1). Except for Johor, populations that represent each state have only a haplotype with no sharing haplotype detected. The haplotypes are as follows: Perak (Hap 4), Terengganu (Hap 5), Kelantan (Hap 3), Ranong Thailand (Hap 2), and Thale Noi (Hap 6).

The nucleotide diversity *P*_*i*_ (t) among the populations was low, ranging from 0.1% to 0.6% (Table 2). The highest *P*_*i*_ (t) value was between Johor and Ranong (0.6%), while the lowest *P*_*i*_ (t) value was between Kelantan and Thale Noi (0.13%). The net nucleotide divergence (*D*_*a*_) among the populations was also low, ranging from 0.1% to 0.9%. The highest *D*_*a*_ value was between Johor and Kelantan populations (0.91%), while the lowest *D*_*a*_ value was between Kelantan and Thale Noi populations (0.13%).

**Figure 3:**
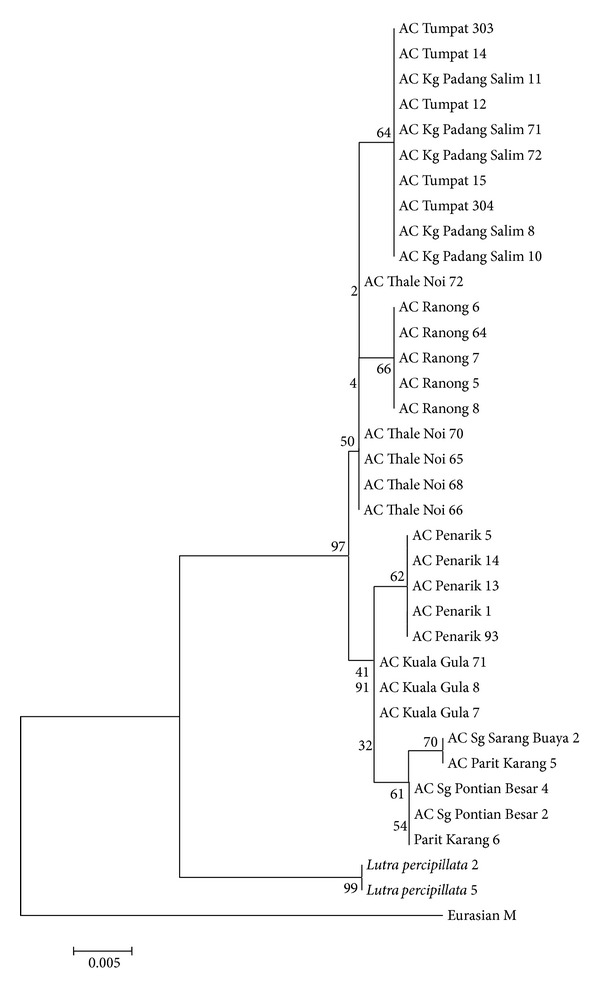
NJ tree topology for studied samples. The number at the branches indicates the bootstrap values for 1000 replications.

**Figure 4:**
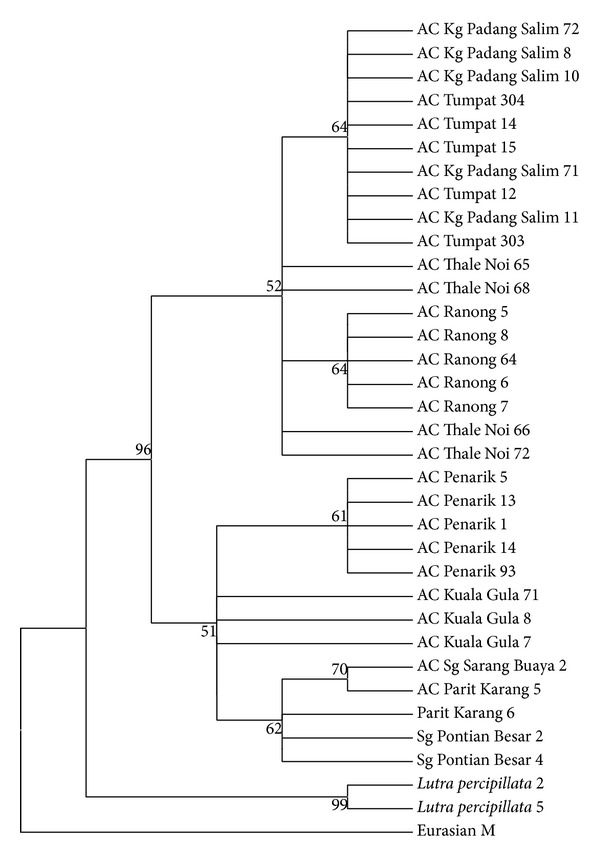
MP tree topology for studied samples. The number at the branches indicated the bootstrap values of 1000 replications.

Phylogenetically, the NJ and MP trees shared the same tree topologies (Figures 3 and 4). From both trees, all otter species were grouped together distinct from the outgroup sample of the Eurasian otter (GenBank). In the main clade (clade A), the* A. cinereus* samples were grouped together in another clade (clade B) distinct from the outgroup samples of* L. perspicillata* (clade C). In the major clade of* A. cinereus* samples were two other clades (clade D and clade E) with 96% bootstrap support. CladeD consisted of samples from Kelantan (Kg Padang Salim and Tumpat) and Thailand (Ranong and Thale Noi). Samples from the two Kelantan populations formed a monophyletic clade distinct from the monophyletic clade of Ranong and Thale Noi. However, the clustering of each group was not supported by high bootstrap values (<70%). In clade E, two subclades formed, also with low bootstrap values. The first subclade consisted of samples from Terengganu (Penarik), and the second subclade consisted of two monophyletic clades from Perak (Kuala Gula) and Johor (Sg Sarang Buaya and Pontian). The tree topologies showed two cluster patterns by population: the east coast Malaysian-southern Thai population (Kelantan, Thale Noi, and Ranong) and the southern, northern, and east coast Malaysian population (Perak, Johor, and Terengganu).

Due to the limited number of haplotypes in each population, a mismatch distribution analysis was not suitable in this study except for the population involving two countries, Thailand and Malaysia. Two populations were observed for mismatch analysis after the haplotypes of each state of each country were merged (Figure 6). The scatterplot of Malay Peninsula population indicated multimodal mismatch distribution from the observed frequencies of pairwise differences among the D-loop sequences and the expected frequencies under the sudden and spatial expansion models. However, Thai population indicated unimodal interpretation of mismatch distribution by following the sudden and spatial expansion models (Figure 6).

Genetic distance analysis was performed using the Kimura 2 Parameter (Table 3). Results showed that the* A. cinereus* samples are greatly distanced from* L. perspicillata* (>0.0309). Among the populations, the samples from Malay Peninsula states are closer to each other and highly distant from Thai population except for Kelantan. Kelantan population was closer to Thai population (0.0044) than to the other Malay Peninsula states (0.0079–0.0100).

**Figure 5:**
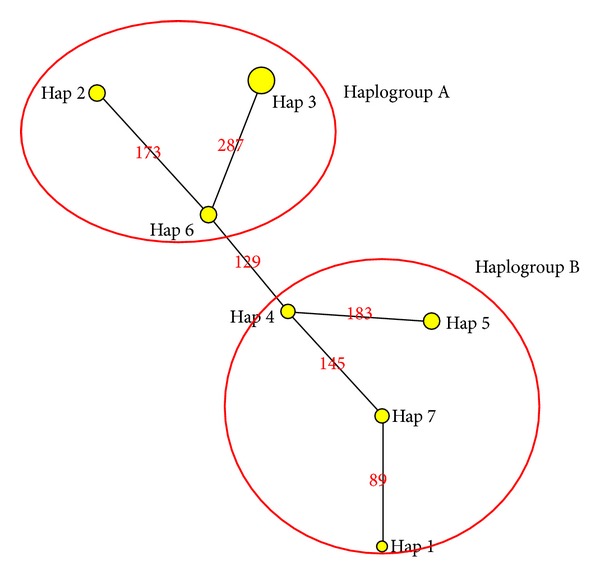
Minimum-spanning network of haplogroup A and haplogroup B.

Differentiation between individual haplotypes within groups was low, with most separated by single base substitutions. The minimum-spanning network that describes the relationships between the population haplotypes is shown in Figure 5. Two distinct networks were observed between haplogroup A (Hap 2, Hap 3, and Hap 6) and haplogroup B (Hap 1, Hap 4, Hap 5, and Hap 7).

## 4. Discussion

DNA from all fecal samples was extracted, amplified, and sequenced successfully. Thirty-three of 48 samples were identified as* A. cinereus* through site polymorphisms. The primer used in this study was useful for mini-DNA barcoding for the three Malaysian otter species. The use of a fresh noninvasive sample source, feces, with the D-loop primer pairs would be suitable for forensic analysis in the future especially for identifying species. As many as 30 informative sites were observed for species-specific differentiation among the three species* L. perspicillata*,* A. cinereus*, and* L. sumatrana*, which represented 7.5% of the total sequence length including some deletions and an insertion in the* L. sumatrana* sequences. The deletion and insertion events in* L. sumatrana* provided the initial idea that* L. sumatrana* differs from the other two species based on the observed DNA barcodes, thus supporting findings by Koepfli et al. [14]. Our finding also contradicts the previous phylogeny classification based on the morphological characteristics of the grouping of* L. sumatrana* and* L. perspicillata* in one group distinct from* A. cinereus* [19–21]. However,* L. perspicillata ta* and* A. cinereus* have significant differences in morphological characteristics and ecological aspects such as body mass and diet selection [58]. According to Koepfli et al. [14], based on continuous observation, there are some possible explanations for this grouping. (a) Hybridization between the two species may occur and is not impossible since the two species have the same chromosome number, 2*n* = 38. (b) The species share a similar brain structure with a bigger rear sigmoid gyrus that gives higher tactile sensitivity, influencing similarities in foraging activity for both species. (c) Based on extinct* Lutrogale paleoleptonyx* and* Lutrogale robusta* fossils found in Java, Indonesia, both* Lutrogale *species have teeth structures suitable for eating shelled foods, a diet similar tothat of* A. cinereus*. In short, the DNA barcode of the three species can be used for species identification studies.

Haplotype analysis showed that there are seven haplotypes, and only Johor state has two haplotypes. The analysis showed that the* A. cinereus* samples from each population are highly endemic based on the partial D-loop sequences. This was supported by the low nucleotide diversity among populations valued below 0.6%. The nucleotide divergence *D*_*a*_ was also observed to be low among populations with the highest *D*_*a*_, between Johor and Ranong (0.91%), which was proportional to the largest distance among other states.

Phylogenetically, all* A. cinereus* samples were excluded from the outgroups, the Eurasian otter and* L. perspicillata*. In the major* A. cinereus* clade, two subclades formed. The first subclade grouped all Malay Peninsula samples except for the samples from Kelantan. The second subclade grouped Kelantan samples with Thai samples. Kelantan samples were outliers in this study as Kelantan was only peninsular state grouped with Thai samples. Based on distance observation, Terengganu is closer to Kelantan, without any major natural barrier preventing gene flow between the two states. Both states are located on the east coast of Malaysia and are farther away from the other states examined in this study. However, genetic distance analysis supported the close relationships of Thai and Kelantan samples compared to the samples from Terengganu and other Malaysian states. Our minimum-spanning network also showed that the samples from Kelantan were grouped as a network with Thai populations. The other Malay Peninsula populations formed another group.

Based on the mismatch analysis for Malay Peninsula states, the observed pattern showed a disrupted flow chart and differed from the simulated pattern. However, the same analysis conducted for Thai populations showed that the observed pattern followed the simulated pattern significantly. These patterns suggested that a disruption exists among the population of Malay Peninsula, which is supported by the outlier formation of Kelantan samples in this study. No previous population study stated any close relationship within Malay Peninsular samples and with Thai populations in* A. cinereus*.

According to Lariviere [22],* A. cinereus* inhabits Bangladesh, Bhutan, Borneo, Brunei, southern China, southern India, Indonesia, Java, Karimun Island, Laos, Malay Peninsula, Myanmar, Palawan, Philippines, Sumatra, Thailand, and Vietnam. This species is likely extinct in Hong Kong and Singapore [28, 29]. Based on previous studies, several subspecies of* A. cinereus* have been recognized. Corbet and Hill [59] described three subspecies of* A. cinereus* in their book* The Mammals of the Indo-Malayan Region: A Systematic Review*. The three subspecies are* A. c. fulvus* (distributed in Vietnam),* A. c. wurmbi* (distributed in East Java), and* A. c. nirnai* (distributed in India). Lariviere [22] also described another two subspecies based on Harris's [60] recognition,* A. c. cinereus* and* A. c. concolor *(location not mentioned).

To date, there is no record of a Malay Peninsula subspecies. However, there is a subspecies named for the neighboring country, Thailand. According to Lekagul and McNeely [61], the subspecies of* A. cinereus *otter in Thailand is* A. c. cinerea*, recognized by Illiger (1815). Since this species is distributed mainly in Southeast Asia,* A. c. cinerea* could be distributed on Thai mainland and then migrated beyond the country's border to the adjacent Malaysian states Kelantan, Kedah, and Perlis. The phylogenetic trees and mismatch distribution analysis in this study supported this cross-country migration. Results also showed that Kelantan-Thai subspecies is completely genetically distinct from* A. cinereus* of the other states of Malay Peninsula.

**Figure 6:**
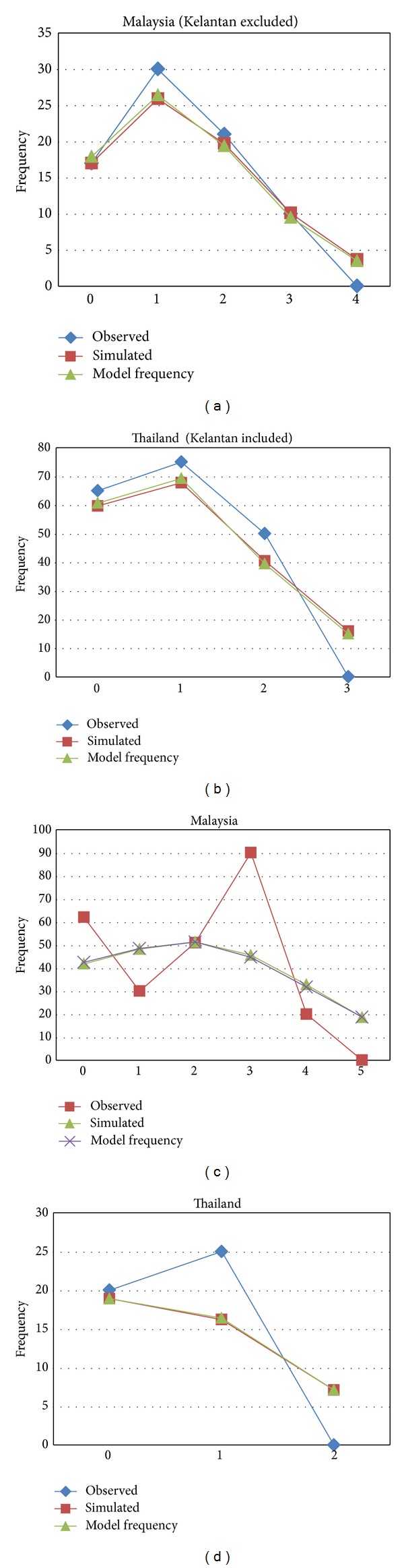
Mismatch distribution of the observed frequencies of pairwise differences among D-loop sequences and expected frequencies under the sudden and spatial expansion models, including and excluding Kelantan population for each country.

For further confirmation, another mismatch analysis was performed by excluding Kelantan population from Malay Peninsula (group 1) and combining it with the populations from Thailand (group 2). Results showed that the observed mismatch distribution pattern for both groups followed the simulated suggested patterns (Figure 6). These patterns suggested that the otters sampled from Kelantan are closer or most likely belong to Thai population and are quite different from the samples from other states. This finding shows that Thai subspecies* A. c. cinerea* may have migrated to Kelantan from Thai mainland. Genetically, these results also suggested the endemic exclusion of Malay Peninsula populations and distinction from Kelantan-Thai populations.

In this study, based on several population analyses, we suggest that Malay Peninsula small-clawed otter is different from Thai subspecies* A. cinereus cinerea*. We also suggest the classification of the new subspecies of Malay Peninsula small-clawed otter named* A. cinereus kecilensis. *This new subspecies might range from the southern to the northern part of Malay Peninsula. However, there was no confirmation that Thai subspecies occurs in the two states located next to Thailand, Perlis and Kedah.

In conclusion, fecal samples were a source for identifying species, which thus supports noninvasive sampling for genetic analysis. The primer pair used in this study was also significant for DNA barcoding for the three Malaysian species* L. perspicillata*,* L. sumatrana*, and* A. cinereus*. This study suggested new classification of Malay Peninsula small-clawed otter,* A. cinereus kecilensis. *However, additional samples from other states of Malay Peninsula are needed to confirm the range of Thai subspecies in Malaysia and the range of* A. cinereus kecilensis* in the northern states.

## Figures and Tables

**Table 1:** List of samples.

Species	Haplotype	Sample	Locality	State
*A. cinereus *	Hap 7	Sg_Sarang_Buaya_2	Sg Sarang Buaya	Johor, Malaysia
*A. cinereus *	Hap 7	Sg_Pontian_Besar_2	Sg Pontian besar	Johor, Malaysia
*A. cinereus *	Hap 1	Sg_Pontian_Besar_4	Sg Pontian besar	Johor, Malaysia
*A. cinereus *	Hap 1	Parit_Karang_5	Parit Karang	Johor, Malaysia
*A. cinereus *	Hap 1	Parit_Karang_6	Parit Karang	Johor, Malaysia
*A. cinereus *	Hap 4	Kuala_Gula_71	Kuala Gula	Perak, Malaysia
*A. cinereus *	Hap 4	Kuala_Gula_7	Kuala Gula	Perak, Malaysia
*A. cinereus *	Hap 4	Kuala_Gula_8	Kuala Gula	Perak, Malaysia
*A. cinereus *	Hap 5	Penarik_5	Penarik	Terengganu, Malaysia
*A. cinereus *	Hap 5	Penarik_1	Penarik	Terengganu, Malaysia
*A. cinereus *	Hap 5	Penarik_93	Penarik	Terengganu, Malaysia
*A. cinereus *	Hap 5	Penarik_13	Penarik	Terengganu, Malaysia
*A. cinereus *	Hap 5	Penarik_14	Penarik	Terengganu, Malaysia
*A. cinereus *	Hap 3	Kg_Padang_Salim_71	Kg Padang Salim	Kelantan, Malaysia
*A. cinereus *	Hap 3	Kg_Padang_Salim_72	Kg Padang Salim	Kelantan, Malaysia
*A. cinereus *	Hap 3	Kg_Padang_Salim_8	Kg Padang Salim	Kelantan, Malaysia
*A. cinereus *	Hap 3	Kg_Padang_Salim_11	Kg Padang Salim	Kelantan, Malaysia
*A. cinereus *	Hap 3	Kg_Padang_Salim_10	Kg Padang Salim	Kelantan, Malaysia
*A. cinereus *	Hap 3	Tumpat_304	Tumpat	Kelantan, Malaysia
*A. cinereus *	Hap 3	Tumpat_303	Tumpat	Kelantan, Malaysia
*A. cinereus *	Hap 3	Tumpat_12	Tumpat	Kelantan, Malaysia
*A. cinereus *	Hap 3	Tumpat_14	Tumpat	Kelantan, Malaysia
*A. cinereus *	Hap 3	Tumpat_15	Tumpat	Kelantan, Malaysia
*A. cinereus *	Hap 2	Ranong_64	Ranong	Thailand
*A. cinereus *	Hap 2	Ranong_5	Ranong	Thailand
*A. cinereus *	Hap 2	Ranong_6	Ranong	Thailand
*A. cinereus *	Hap 2	Ranong_7	Ranong	Thailand
*A. cinereus *	Hap 2	Ranong_8	Ranong	Thailand
*A. cinereus *	Hap 6	Thale_Noi_70	Thale Noi	Thailand
*A. cinereus *	Hap 6	Thale_Noi_65	Thale Noi	Thailand
*A. cinereus *	Hap 6	Thale_Noi_66	Thale Noi	Thailand
*A. cinereus *	Hap 6	Thale_Noi_68	Thale Noi	Thailand
*A. cinereus *	Hap 6	Thale_Noi_72	Thale Noi	Thailand
*L. perspicillata *		*L. perspicillata*_2	Alai	Melaka, Malaysia
*L. perspicillata *		*L. perspicillata*_5	Alai	Melaka, Malaysia
		Eurasian		GenBank

**Table 2:** Nucleotide diversity *P*_*i*_ (t) and nucleotide divergence (*D*_*a*_) between populations.

Populations	Nucleotide diversity *P*_*i*_ (t)	Nucleotide divergence (*D*_*a*_)
Johor-Ranong	0.00593	0.00909
Johor-Kelantan	0.00493	0.00912
Johor-Thale Noi	0.00430	0.00616
Johor-Terengganu	0.00431	0.00618
Johor-Perak	0.00284	0.00324
Ranong-Kelantan	0.00264	0.00554
Ranong-Thale Noi	0.00138	0.00248
Ranong-Terengganu	0.00462	0.00462
Ranong-Perak	0.00462	0.00462
Kelantan-Thale Noi	0.00132	0.00132
Kelantan-Terengganu	0.00396	0.00831
Kelantan-Perak	0.00223	0.00223
Thale Noi-Terengganu	0.00308	0.00554
Thale Noi-Perak	0.00155	0.00290
Terengganu-Perak	0.00155	0.00290

**Table 3:** Genetic distance analysis among populations for *A. cinereus* D-loop sequences.

	Johor	Perak	Terengganu	Kelantan	Thailand
Johor					
Perak	0.0041				
Terengganu	0.0071	0.0029			
Kelantan	0.0100	0.0079	0.0088		
Thailand	0.0085	0.0069	0.0094	0.0044	
*L. perspicillata *	0.0353	0.0324	0.0353	0.0324	0.0309
